# Short-term results of endoscopic calcaneoplasty and retrocalcaneal bursectomy for insertional Achilles tendinopathy

**DOI:** 10.1097/MD.0000000000035349

**Published:** 2023-10-06

**Authors:** Se-Hwan Lee, Kwang-Bok Lee

**Affiliations:** a Department of Orthopedic Surgery, Research Institute of Clinical Medicine of Jeonbuk National University, Biomedical Research Institute of Jeonbuk National University Hospital, Jeonbuk National University Medical School, Jeon ju, South Korea.

**Keywords:** Achilles tendinopathy, endoscopic calcaneoplasty, heel pain, retrocalcaneal bursitis

## Abstract

Although open surgery has traditionally been used as a surgical treatment for insertional Achilles tendinopathy, there is a possibility of serious complications (avulsion, scarring, contracture, sensory changes, and infection) due to the anatomical characteristics of the area. Endoscopic surgery has some advantages due to the smaller incision needed. The purpose of this study was to evaluate the effectiveness of endoscopic surgery in insertional Achilles tendinopathy. Twelve patients (15 feet) who underwent endoscopic surgery between 2015 and 2021 were included in this study. Clinical results were evaluated before and after surgery by visual analog scale (VAS) and, Ogilvie-Harris scores and complications. For radiological evaluation, the Fowler-Philip angle, and positive parallel pitch line were measured. VAS scores decreased from 7.6 preoperatively to 2.3 at the last postoperative follow-up, and Ogilvie–Harris values showed excellent results in 5 cases, good results in 8 cases, and fair results in 2 cases. In the radiographic results, there was no bone prominence above the Pavlov calcaneus pitch line in any case, and the Fowler-Philip angle decreased from an average of 57.5 degrees to 50.2 degrees. Only 1 patient underwent reoperation due to the recurrence of symptoms 33 months after the first surgery. After the second operation, the VAS score decreased to 3 points. No complications occurred. Endoscopic surgery is an effective and minimally invasive procedure, showing fewer complications and similar satisfaction as the open procedure. Therefore, it can be a good treatment option for patients with insertional Achilles tendinopathy as it provides the patient with a quick return to daily life.

## 1. Introduction

Pain in the posterosuperior portion of the calcaneus is a common symptom reported in about 6% of the total population.^[1]^ It may be caused by insertional or non-insertional Achilles tendinitis, retrocalcaneal bursitis, Haglund’s deformity, or inflammation of an adventitious bursa between the Achilles tendon and the skin.^[2–4]^ Retrocalcaneal bursitis can occur by repeated stimulation or the presence of Haglund’s deformity, which may lead to posterior heel pain.^[5]^ Haglund’s deformity is defined as a bony prominence in the posterosuperior tuberosity of the calcaneus.^[4]^ This bony prominence increases pressure on the hindfoot lesion. In insertional Achilles tendinopathy, pain is felt in the central region of the attachment. It mainly occurs in the 3 to 4 cm proximal part of the Achilles tendon attachment. Non-insertional Achilles tendinopathy is more common than insertional Achilles tendinopathy, and the lesion is located 2 to 6 cm above the attachment site.

Treatment in the acute phase is performed to control inflammation. Complex treatments such as shoe wear modification, heel lift, stretching of the gastrocnemius-soleus complex, anti-inflammatory medication, steroid injection, and shockwave therapy can also be performed.^[6,7]^

If there is no response to 4 to 6 months of conservative treatment, surgical treatment may be considered. The goal of treatment is to reduce pressure on the Achilles tendon. Open surgery has been the conventional treatment method. A longitudinal incision is made in the Achilles tendon from above the calcaneus to remove the tissue around the inflamed tendon and bony prominence. Depending upon the degree of removal, reinforcement or relocation is performed using either the flexor hallucis longus tendon or the peroneal brevis tendon. Many studies have reported good results from such open surgery.^[8–16]^ However, surgical wound complications are frequently reported due to the anatomical characteristics of poor blood circulation in the heel region. In addition, complications such as avulsion fracture due to the excessive removal of the posterior upper calcaneus or Achilles tendon contracture due to prolonged immobilization have been reported. Scarring may also occur due to the relatively large skin incision, and pain and sensory changes around the scar may appear.^[9,17–19]^

In contrast, endoscopic surgery requires a smaller incision than open surgery, so it is possible to reduce infection and pain at the wound site, which may occur after surgery. In addition, because rehabilitation exercises can be initiated soon after surgery, complications that may occur due to immobilization can be minimized, and patients may be able to return to daily life more quickly. Therefore, endoscopic surgery may be a therapeutic alternative for Achilles tendinitis, retrocalcaneal bursitis, and Haglund’s deformity.

The purpose of this study was to prove that minimally invasive surgery can be a good alternative treatment by examining patient results after endoscopic procedures for Achilles tendinopathy originating from various causes.

## 2. Material and methods

### 2.1. Patients selection

This study research has been approved by the IRB of the authors affiliated institutions. We retrospectively reviewed the records from June 2015 to June 2021, a total of 12 patients (15 feet) who were diagnosed with Achilles tendinopathy underwent endoscopic calcaneoplasty, retrocalcaneal bursectomy, and debridement. The patients consisted of 5 males and 7 females, with an average age of 46 years (range, 23–63 years). The average morbidity period before surgery was 22.5 months (range, 5–50 months), and although conservative therapy was performed for at least 6 months before surgery, there was no improvement in symptoms. On physical examination, there was tenderness in the heel and Achilles tendon attachment site, and pain during dorsiflexion of the foot. The hindfoot axis was normal and there was no varus deformity. There was no history of gout or rheumatoid arthritis in any patient.

**Figure 1.**
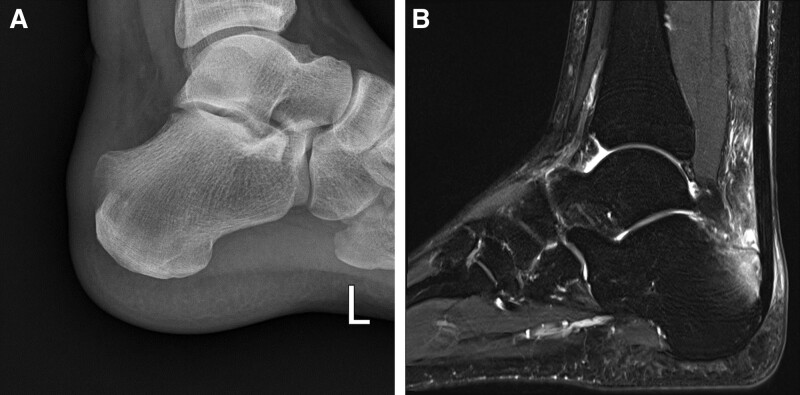
A 27-year-old male with insertional Achilles tendinopathy. The preoperative ankle lateral plain radiogram shows Haglund’s deformity. MRI shows the retrocalcaneal bursa and mid-substance, the insertion area of the Achilles tendon, and thickening of the Achilles tendon. MRI = magnetic resonance imaging.

The preoperative radiographic findings showed that the hindfoot axis that measured the angle between the tibial shaft axis and the calcaneal axis was normal, and bone protrusion was observed above the calcaneal tubercle on the lateral view. On the lateral radiographs, all patients showed bone prominence above the parallel pitch line (PPL) and the Fowler-Philip angle (FPA) was measured to be an average of 57.5 degrees (range, 48 to 70 degrees). Magnetic resonance imaging was performed in all patients, showing retrocalcaneal bursitis and an increase in signal intensity in the posterior calcaneal bone above the Achilles tendon. (Fig. 1)

### 2.2. Surgical technique

For surgery, the patient was placed in the prone position under general anesthesia, and the knee joint was flexed about 30 degrees by a tourniquet inflated at the thigh. The surgeon sat on the proximal part of the patient’s foot and performed the operation. First, to obtain the lateral portal, the needle was placed on the posterior and superior side of the calcaneus just to the lateral side of the Achilles tendon and placement confirmed using an image intensifier. After confirming the position of the needle, a vertical skin incision of about 0.5 cm was made. The soft tissue was dissected while paying attention to sural nerve damage and the bursa of the posterior calcaneus was approached. The medial portal was made while checking with a scope through the lateral portal. The risk of iatrogenic Achilles tendon damage was reduced because the Achilles attachment site was located more distal than the lateral portal.

**Figure 2.**
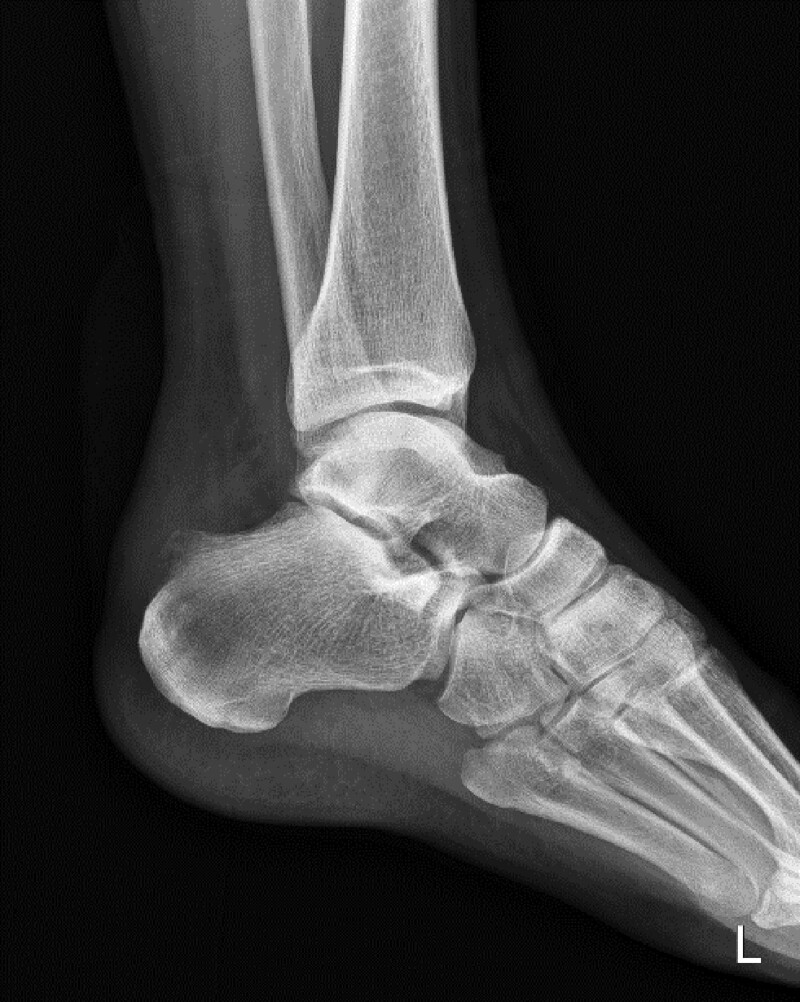
The postoperative ankle lateral plain radiogram shows the removal of Haglund’s deformity.

Adipose tissue in the anterior of the Achilles tendon and bursa tissue suspected of being inflamed were removed with a shaver using 2.7-mm 30° video arthroscope, through the medial portal, and the posterior calcaneus and Achilles tendon were identified. The extent of the bone resection was confirmed with an image intensifier after marking the resection site with 2 needles, and resection was performed using a burr at the apex of the posterosuperior bone of the calcaneus. Resection was performed from the medial to the lateral side, the attachment of the Achilles tendon was confirmed by the distal part, and damage was minimized. (Fig. 2) The degree of resection was checked using an image intensifier, and finally, the remaining lesion site was identified by dorsiflexion of the ankle joint as much as possible. At the end of the surgery, the skin incisions were sutured, and a compression dressing was applied. No short leg splint was applied and non-weight bearing ambulation was performed for 1 week after surgery. One week after surgery, the patient started to walk without restriction, and the patient was instructed not to put pressure on the heel. Exercise was started 6 weeks after surgery.

### 2.3. Assessment

Clinical outcomes were evaluated using a subjective scale for pain visual analog scale (VAS) and Ogilvie-Harris scores^[20]^ and complications. Ogilvie-Harris values evaluated pain, edema, stiffness, limping, and activity as excellent, good, average, and poor and the lowest evaluation score was used as the overall evaluation score. VAS pain scores were collected preoperatively and 7 days, 1, 3, 6, 9, and 12 months and at the last follow-up. Ogilvie-Harris scores and complications were investigated preoperatively and at the 12-month follow-up.

**Figure 3.**
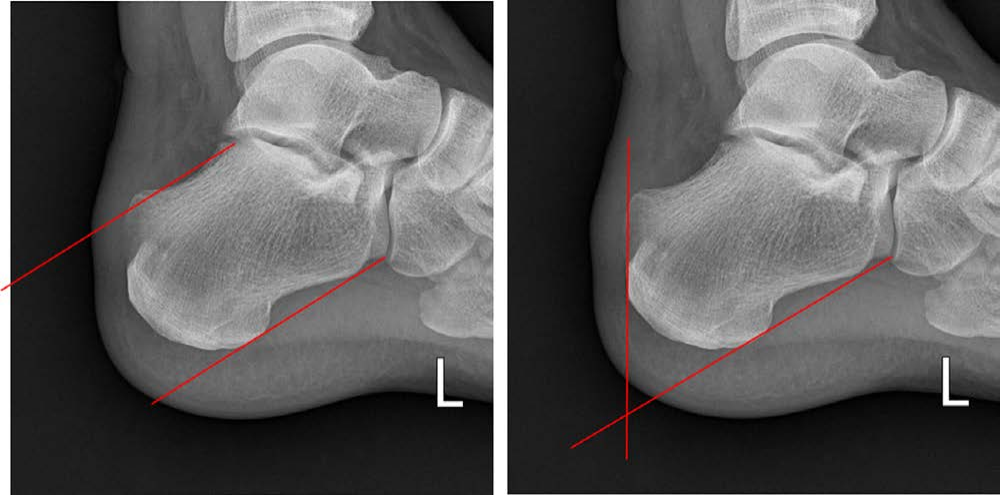
(A) Parallel pitch line. Inferior line tangential to the posterior prominence and the inferior margin of the calcaneocuboid joint. Superior line parallel to inferior line beginning at posterior margin of subtalar joint. Bone above superior line is abnormal. Figure 3 (B) Fowler-Philip angle. Inferior line tangential to the posterior prominence and the inferior margin of the calcaneocuboid joint. Superior line tangential to posterior calcaneal surface.

For radiological evaluation, the presence of bone above the PPL^[21]^ and the FPA^[22]^ (Fig. 3) were measured on lateral radiographs after surgery and at the final follow-up.

Pre- and postoperative results were analyzed using IBM SPSS Statistics version 24.0 (SPSS Inc., IBM, Chicago, IL). Statistical analysis of VAS pain score changes was performed utilizing the Wilcoxon signed-rank test because it not satisfied normality. FPA was evaluated by the paired t-test. *P* values of < .05 were considered statically significant.

The average follow-up period after surgery was 10 ± 8.68 months, and lateral radiographs were taken at each follow-up. The average operation time was 74 ± 22.35 minutes. The operation time decreased with increases in the learning curve, so the most recent 7 cases took 61 ± 16.65 minutes. The average length of the hospital stay was 7 ± 1.42 days (Table 1).

**Table 1:** Patient demographics.

No.	Age	Sex	BMI	Smoking	Symptom duration (mo)	Follow-up (mo)	Operative time (min)	VAS (Pre/Last f/u)	O-H score
1	45	F	24.6	No	36	8	106	8/2	Good
2	51	F	23.4	No	12	5	86	8/3	Good
3	51	F	23.4	No	12	5	86	8/1	Excellent
4	60	M	24.0	No	6	37	72	8/5	Fair
5	55	M	26.4	Yes	24	6	128	8/2	Good
6	55	F	29.0	No	5	22	70	8/1	Good
7	42	M	33.2	Yes	48	10	75	7/2	Good
8	63	F	20.8	No	12	3	59	9/4	Fair
9	57	F	31.9	Yes	12	13	83	6/1	Good
10	26	F	26.4	No	5	5	39	5/3	Good
11	23	M	23.2	No	48	10	55	8/2	Excellent
12	23	M	23.2	No	50	7	88	8/2	Excellent
13	63	M	24.0	Yes	15	2	55	8/3	Good
14	27	M	29.8	No	3	6	48	8/1	Excellent
15	41	F	24.3	No	7	6	63	8/3	Excellent

BMI = body mass index, O-H score = Ogilvie–Harris score, pre/last f/u = preoperative/last follow-up, VAS = visual analog scale.

On the arthroscopic findings, retrocalcaneal bursitis was observed in all patients, so debridement was performed, and calcaneoplasty was also performed in all patients.

## 3. Results

In the clinical results, VAS scores decreased from 7.6 preoperatively to 2.7 on the 7th day after surgery and were maintained at 2.3 ± 0.17 at 1, 3, 6, and 12 months of follow-up. The mean follow-up duration was 9.7 months (Range 2–37months) The Ogilvie–Harris score showed excellent results in 5 cases, good results in 8 cases, and average results in 2 cases. In the radiographic results, there was no presence of bone above the PPL in all cases, and the FPA decreased from an average of 57.5 degrees to 50.2 degrees (Table 2).

**Table 2:** Comparison between preoperative and last follow-up VAS scores and radiographic parameters.

	Pre-OP	7 days	1 months	3 months	6 months	9 months	12 months	Last follow-up	*P* value
VAS	7.67	2.7	2.4	2	2.2	2.1	2.5	2.1	.001
	Average for 1 yr							2.2	
Fowler-Philip angle	57.53							50.20	<.001
Pavlov line	+							−	

VAS = visual analog scale, Pre-OP = preoperative.

In 1 patient, pain decreased after the first operation, but increased again 6 months after surgery, and reoperation was performed 33 months after the index surgery. After the reoperation, the VAS score decreased to 3 points. There were no complications such as postoperative neurovascular complications, wound infection, or delayed rupture of the Achilles tendon.

## 4. Discussion

Hindfoot pain around the Achilles tendon can have a variety of causes. In some cases, the terminology is ambiguous, and the pathology overlaps and often appears as clinical symptoms, so a careful diagnosis is necessary for determining the appropriate treatment method. In this study, we analyzed patient groups with insertional Achilles tendinitis, retrocalcaneal bursitis, and Haglund’s deformity.

In the case of insertional Achilles tendinitis, deformation due to mucoid degeneration, bleeding, necrosis, and calcification that occurs within the tendon of the attachment area appear, and patients complain of pain at the attachment site. In retrocalcaneal bursitis, an inflammation that occurs at a location similar to insertional Achilles tendinitis causes hindfoot pain due to continuous pressure or repeated overuse. Haglund’s deformity is an anatomical change that shows bony prominence in the posterosuperior tuberosity of the calcaneus, resulting in increased hindfoot pressure and continuous stimulation.

In this study, endoscopic treatment was performed for all 15 cases in 12 patients. In all patients, the lesion was approached through a 0.5 cm sized skin incision on the medial and lateral sides from about 2 to 4 cm above the proximal part of the Achilles tendon insertion site. Retrocalcaneal bursectomy was performed, and calcaneoplasty was performed under an image intensifier. In addition, the inflammatory tissue around the surrounding Achilles tendon was removed. This endoscopic treatment method was first described by Van Dijk et al^[23]^. As a result of follow-up for an average of 3.9 years in 21 cases, excellent or good results were reported in 19 cases (90.5%). Jerosch et al^[24]^ reported excellent or good results in 75 cases (92.6%) after an average follow-up of 35.3 months in 81 cases. As a result of follow-up for an average of 7.6 months in 15 cases in this study, excellent or good results were obtained in 13 cases (86.7%). Also, the average VAS score decreased to 2.7 on the 7th day after surgery, and the pain level was similar until the last follow-up (Table 2). In contrast, in the case of open surgery, the clinical results of the surgery showed a lot of deviation. According to Wiegerinck et al^[25]^, in 3 studies with open surgery, 30 to 42% of the patients showed no change or worse results,^[9,12,15]^ and in another 6 other studies, 81% to 95% of the patients had excellent or good results.^[10,11,13,14,16,26]^ In the clinical results, the endoscopic treatment showed similar or better results than most open surgeries and could be considered to achieve reliable results. First, since only a small wound was left, pain and nerve damage around the surgical wound could be minimized. Second, decreases in satisfaction due to complications such as Achilles tendon contracture were minimized by starting joint movements and weight bearing sooner than in open surgery. Finally, compared to open surgery, endoscopic surgery has the advantage of being able to remove sufficient calcaneus bony spurs and inflamed tissue around the retrocalcaneal bursa by using both the medial and lateral viewing portals.

Among 15 patients in this study, no complications were reported. In the case of open surgery, according to Wiegerinck et al^[25]^, major complications such as tendon rupture or deep infection were reported in 397 to 17 cases (4.3%), and minor complications such as superficial infection or scar tenderness were reported in 160 cases (45%). In the case of endoscopic surgery, the frequency was very low, with 1 major complication (0.7%) and 2 cases (1.3%) with minor complications out of 150 cases. The posterior heel part is anatomically weak in blood flow, and this is one of the most frequent locations of wound infections after surgery. In addition, the surgical wound itself can become a large scar, causing discomfort as it is an area that can be continuously stimulated by wearing shoes. Compared to open surgery, endoscopic surgery can minimize complications with a small wound of about 0.5 cm in size.

In postoperative rehabilitation, in this study, non-weight bearing and restrictions in joint range of motion were imposed for about 1 week after surgery, and it was possible for the patients to return to daily life 1 week after surgery. In the case of open surgery, the period of non-weight bearing was approximately 2 to 3 weeks to 6 to 8 weeks.^[25]^ Hindfoot pain occurs frequently from repeated use and in athletes. Thus, if it is necessary to limit the patient’s daily life for a long time, this can become an obstacle in deciding on the treatment for a patient who needs surgery. In this respect, endoscopic treatment can be a more easily recommended treatment method for patients than open surgery.

This study also had some limitations. First, there was no direct comparison with open surgery as it was performed by a single surgeon. While our study has confirmed the advantages of endoscopic calcaneoplasty through comparison with other studies on open surgery, there is a limitation that the surgeon’s technique could not be excluded from the study result. Second, there was a small number of patients and long-term follow-up was not achieved. To minimize the impact of confounding factors on our observations, we made efforts to exclude patients with anatomic deformities, gout, and rheumatoid arthritis, all of which are known to affect heel pain. As a result, we aimed to observe the effect of the surgery as objectively as possible. Therefore, the rationale for endoscopic therapy should be supported by accumulating more surgical cases and long-term follow-ups.

## 5. Conclusion

Our study suggests that endoscopic bursectomy and calcaneoplasty may yield positive outcomes in terms of pain relief and radiological improvements for patients with Achilles tendinopathy who do not respond to conservative treatment. Furthermore, our study revealed that endoscopic surgery yielded similar surgical outcomes with less scarring and a shorter rehabilitation period when compared to the results reported in previous studies on open surgery. Therefore, endoscopic surgery may be a good alternative to open surgery.

## Acknowledgments

This research was supported by the Korea Health Technology R&D Project through the Korea Health Industry Development Institute (KHIDI), funded by the Ministry of Health & Welfare, Republic of Korea (grant number: HR22C1832).

## Author contributions

**Conceptualization:** Kwang-Bok Lee.

**Data curation:** Se-Hwan Lee.

**Formal analysis:** Se-Hwan Lee.

**Funding acquisition:** Kwang-Bok Lee.

**Investigation:** Kwang-Bok Lee.

**Methodology:** Kwang-Bok Lee.

**Project administration:** Kwang-Bok Lee.

**Resources:** Kwang-Bok Lee.

**Supervision:** Kwang-Bok Lee.

**Writing – original draft:** Se-Hwan Lee.

**Writing – review & editing:** Kwang-Bok Lee.
